# Migraine symptoms, healthcare resources utilization and disease burden in a large Polish migraine cohort: Results from ‘Migraine in Poland’—a nationwide cross-sectional survey

**DOI:** 10.1186/s10194-023-01575-4

**Published:** 2023-04-11

**Authors:** Marta Waliszewska-Prosół, Marcin Straburzyński, Ewa K. Czapińska-Ciepiela, Magdalena Nowaczewska, Anna Gryglas-Dworak, Sławomir Budrewicz

**Affiliations:** 1 Department of Neurology, Wrocław Medical University, Borowska 213 Str., 50-556 Wrocław, Poland; 2 Department of Family Medicine and Infectious Diseases, University of Warmia and Mazury, Olsztyn, Poland; 3 Epilepsy and Migraine Treatment Centre, Kraków, Poland; 4 Department of Otolaryngology, Head and Neck Surgery and Laryngological Oncology, LudwikRydygier Collegium Medicum in Bydgoszcz, Nicolaus Copernicus University in Toruń, Bydgoszcz, Poland; 5 Headache Center, Wrocław, Poland

**Keywords:** Headache, Quality of life, Abortive treatment, Preventive treatment, Prophylactic treatment, Aura, ICHD-3

## Abstract

**Background:**

The Migraine in Poland study is the first large scale nationwide cross-sectional online survey of symptoms, approaches to management, treatment patterns, quality of life, and sociodemographic characteristics of the Polish migraine patients’ cohort, conducted from August 2021 to June 2022.

**Methods:**

A cross-sectional online survey was designed based on the American Migraine Prevalence and Prevention (AMPP) Study. Participants were recruited through broad advertisement through various channels. The survey included questions allowing for the diagnosis of migraine without aura (MwoA) based on the third edition of the International Classification of Headache Disorders (ICHD-3). Moreover, the questionnaire assessed sociodemographic and headache features, comorbidities, consultation rates with medical professionals, as well as the use of abortive or preventive treatment, including non-pharmacological methods, psychological symptoms and the burden of migraine.

**Results:**

A structured online questionnaire was submitted by 3225 respondents aged 13 to 80 (mean age 38.9, 87.1% women). In this group 1679 (52.7%) of participants fulfilled ICHD-3 diagnostic criteria for MwoA, which was in most cases (88.3%) confirmed by a medical professional in the past. In this group the average number of monthly headache days was 4.7, while 47.8% of participants had at least 4 migraine days per month. Mean Migraine Disability Assessment score was 42.65 (median 32).

Among MwoA respondents, 1571 (93.6%) had consulted their headache with a medical professional in the past – mostly neurologists (*n* = 1450 (83.4%) and primary care physicians (*n* = 1393 (82.9%). In the MwoA cohort, 1553 (92.5%) of participants declared the current use of some form of treatment, although only 193 (11.5%) respondents were currently on preventive medications. The most prevalent comorbidities included: chronic rhinitis (37.1%), allergies (35.9%) and low blood pressure (26.9%). Anxiety (20.4%) and depression (21.3%) were highly prevalent among participants.

**Conclusions:**

People with migraine in Poland face similar difficulties as their peers in other countries. Despite relatively high access to neurologist consultations and good diagnosis accuracy, migraine still poses diagnostic and therapeutic difficulties. In this context, migraine undertreatment in Polish population must be underlined, especially in context of high disease burden.

## Introduction

About 320 million people worldwide (11–12% of the world's population) experience migraine [1]. Poland is the European Union’s fifth most populous country. Consequently, it is estimated that over 3.5 million of Poles have migraine, more than 400.000 of whom suffer its chronic form [2–4]. Since migraine affects mostly working-age people, it can be assumed that it entails significant financial costs to both the Polish and European economies due to absenteeism or presenteeism [4, 5]. However, apart from economic consequences, migraine is a major disruptor of patients’ private lives, which stems from the unfounded and stigmatizing idea, ingrained in popular consciousness, that migraine is just a ‘standard’ headache or some form of excuse or fad [6, 7].

Reducing the burden of migraine, especially at a time when tremendous progress has been made in treating the disease, is an important public health goal and a challenge for every country. To achieve this goal, it is important to understand the dynamics of migraine diagnosis and course, treatment patterns, and to identify the significant risks of inappropriate management and treatment [6, 8]. Many countries have been already assessed in this area. In the United States, the AMPPs study provided important evidence for necessary change [9]. In Europe, the Eurolight study characterized migraine and its treatment across some nations [10]. However, the results from studies in other countries cannot be simply extrapolated to every population, especially considering that the disease burden, treatment patterns or even symptomatology may to a large extent depend on local social and educational conditions, as well as healthcare system policies.

The Migraine in Poland study was designed as the first large scale study of Poles with migraine. It aims to assess the current symptom patterns, diagnosis, consultation, treatment and burden of migraine on a large representative sample of Poles.

## Methods

This study was designed as a national cross-sectional online survey. Data collection was scheduled from August 2021 till June 2022 using online questionnaire software (Google Forms). All relevant questions were mandatory for respondents before submitting the data. However, in situations when a particular issue was irrelevant (e.g. menstruation in males), the algorithms implemented in the questionnaire omitted these subjects.

### Ethics

The study was registered as ‘Migraine in Poland – a Web-based Cross-sectional Survey’ in the ClinicalTrials.gov database under the number: NCT05087420. The project was approved by the Commission of Bioethics at the Wrocław Medical University. The introduction to the survey included a detailed description of the study. Participants could begin answering the questions only after confirming they had read this disclaimer. All data was kept confidential and in accordance with data protection regulations, while the anonymity of respondents was maintained.

### Screening and recruitment

To publicize and carry out the recruitment, a multichannel campaign was initiated. Respondents were invited to participate in the study through:

- social media (Facebook, Instagram, Twitter),
- national mass media (radio, television, newspapers, websites),
- employees of Poland’s largest state-owned and private companies,
- state and religious institutions,
- secondary schools and universities,
- scientific societies,
- trade unions and non-governmental organizations,
- outpatient service providers in primary and secondary care.

People of all ages reporting at least one day of headache in the past year were eligible to participate in the survey. Respondents received no compensation and received no other benefits from participating in the study.

### Assessments / study design

The questionnaire was developed on the basis of the validated American Migraine Prevalence and Prevention Study (AMPP) [9], taking into account the differences between the USA and Poland in terms of, among others, access to various forms of therapy.

The survey consisted of the following items:

- 1. Sociodemographic characteristics: age, gender, education, employment status, marital and occupational status, place of residence.
- 2. Headache history and characteristics: headache frequency was assessed by recalling the number of headache days over a 3-month period; age of headache onset; the type of headache was determined according to the ICHD-3 criteria [11]; respondents were asked about the number of experienced headache types and accompanying symptoms. The questionnaire assessed the co-occurrence of visual symptoms and headache, as well as their duration. However, the data on laterality, positive vs negative phenomena and gradual development of symptoms were not assessed (in accordance with the AMPP study questionnaire).
- 3. Healthcare utilization – respondents were asked to recall the details of their physician-confirmed headache-type diagnosis. They were asked about the approximate date of their last medical consultation, as well as how often and with what specialist they consulted their headache.
- 4. Acute and prophylactic treatment – respondents were presented with a table with abortive medications available in Poland (OTC and on prescription). Then they were to indicate how many days a month they used a given medication. Questions regarding prophylactic treatment took into account the duration of its use and its effectiveness. Moreover, this section also included the use of non-pharmacological methods for treating migraines, treatment satisfaction and reasons for changing treatment methods (if any). Due to the large quantity of collected data, detailed results on this part of the survey are to be published in a separate paper.
- 5. Headache burden – the Migraine Disability Assessment Scale (MIDAS) was used as a primary burden assessment. Moreover, the questionnaire collected data on absenteeism and presenteeism due to migraine in the previous 3 months. Psychological symptoms from the previous 2 weeks were evaluated using the Patient Health Questionnaire 9 (PHQ-9), validated for the Polish population [12, 13].
- 6. Comorbidities – participants were asked about their previous diagnosis of disorders, including cardiovascular, psychiatric, neurological, endocrine, autoimmune, allergic, respiratory and gastroenterological diseases. Due to the large quantity of collected data, detailed results on this part of the survey are to be published in a separate paper.

### Statistical methods

The statistical analysis was performed using STATISTICA 11.0 PL software.

We used descriptive statistics to present survey-calculating means and standard deviations (SDs) for continuous outcomes (age, BMI, MHD) and proportions (%) for binary and multinomial outcomes (gender, marital status, education level, employment status, headache symptoms and features, allodynia, psychological symptoms, self-reported diagnosis, consulting status, and use of preventive and OTC medications). The analyses were conducted using Microsoft Excel, version 2019.

## Results

### Basic demographic and social data

During the study, 3225 respondents submitted their answers via a structured online questionnaire: 2809 (87.1%) women, 411 (12.7%) men and 5 (0.2%) non-binary participants. Respondents were from 13 to 80 years old (mean 38.9, median 39). In this group, 15 respondents (0.5%) were aged 13–18.

Respondents with MwoA were employed full-time in 870 (51.8%) cases, part-time in 74 (4.4%), while 193 (11.5%) ran their own business. 152 (9.0%) were students and 191 (11.4%) were unemployed. The remaining respondents were otherwise employed (e.g. in agriculture or voluntary service). Participants worked 8 h a day, 5 days a week (median). MwoA participants lived in a large city in 811 (48.3%) cases, in a medium or small municipality in 496 (29.5%) and in the countryside 223 (13.3%). In MwoA cohort, 1143 (68.1%) respondents were married, 127 (7.6%) divorced and 261 (15.5%) single. Master’s degree or higher education was confirmed by 925 (55.1%) of participants, Bachelor degree by 163 (9.7%) and secondary education by 396 (23.6%).

### Headache characteristics

In the 12 months leading to the study, 3167 (98.2%) participants had at least one episode of headache unrelated to infection or hangover. In the 3 months preceding the study, respondents had on average 19.58 (median 15) headache days. Headache occurring in the previous 7 days was reported by 2335 (73.5%) respondents and by 756 (23.8%) in the previous month. The majority of patients 1621 (51.0%) experienced 2 distinct types of headaches, 930 (29.3%) one type and 626 (19.7%) 3 or more types of headaches.

Migraine without aura (MwoA) diagnosis according to ICHD-3 was confirmed in 1679 (52.7%) of respondents (Table 1). Other diagnoses included: probable MwoA *n* = 961 (30.8%), probable MwA *n* = 690 (21.7%), isolated tension-type headache in *n* = 210 (6.5%) (more than one headache diagnosis was possible). 48 (1.5%) respondents denied having ever experienced headache.

**Table 1:** Participants meeting ICHD-3 diagnostic criteria for MwoA

Criterion	*n* (%)
1.1 ICHD-3 MwoA	
B	2390 (75.1)
C	2660 (83.5)
- C1	2408 (75.6)
- C2	2165 (68.0)
- C3	2325 (73.0)
- C4	2062 (64.8)
D	2316 (72.7)
- D1	1738 (54.6)
- D2	2023 (63.5)
MwoA (B + C + D)	1679 (52.7)
Probable MwoA (2 out of 3 of B/C/D)	961 (30.2)
Chronic migraine	117 (3.7)

*MwoA* Migraine without aura

**Fig. 1:**
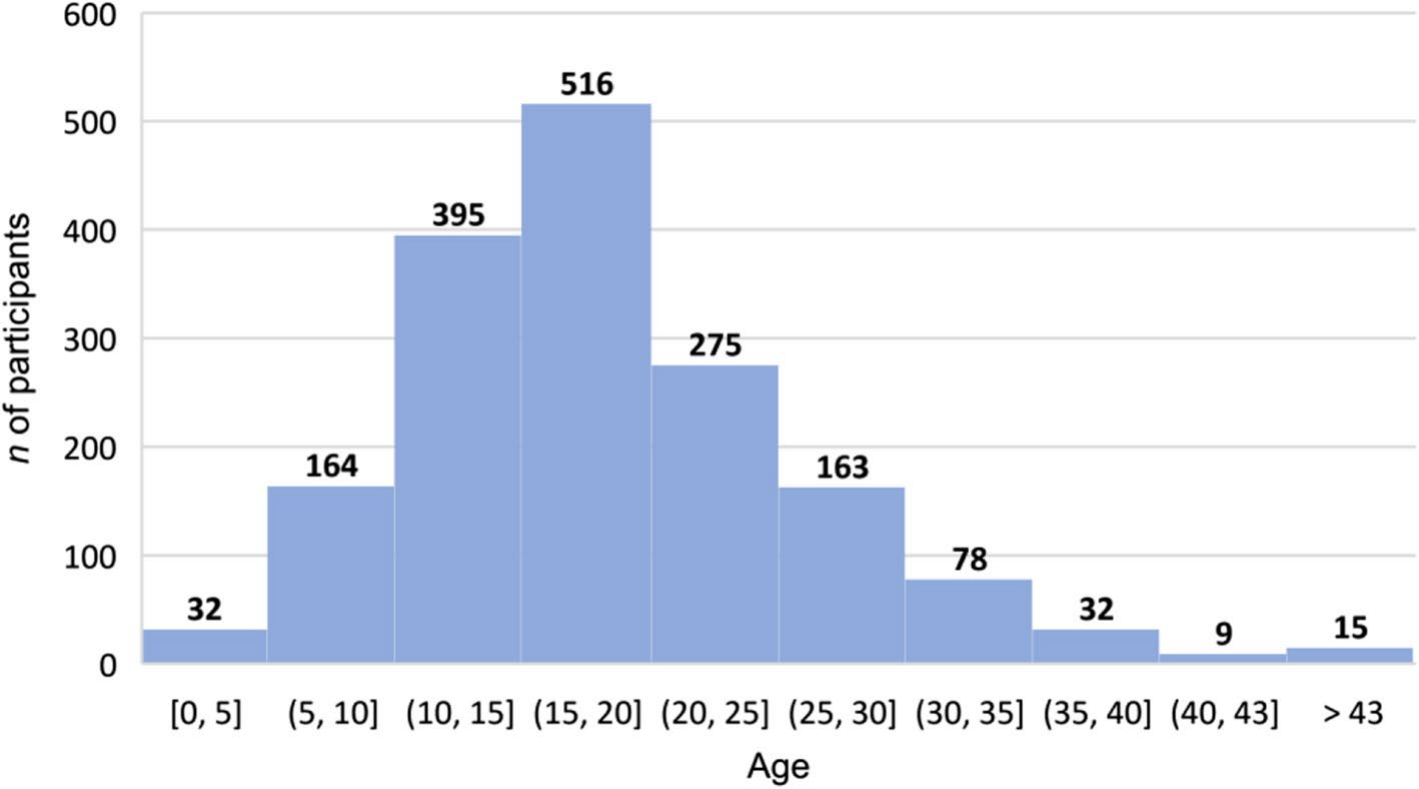
Age at MwoA onset

The first migraine attacks occurred in the second decade of life in the majority of patients (mean age 19.17, median 18) (Fig. 1). MwoA respondents had average 4.69 migraine days in the previous month (median = 3). In this group 802 (47.8%) had at least 4 migraine days per month. In the 3 months preceding the study, headache frequency significantly increased in 349 (20.8%) and decreased in 241 (14.35%) of MwoA participants. Occurrence of migraine attacks with menstruation was reported by 1024 (36.4%) of women.

Spots, stars, lines, flashing lights, zigzag patterns, or "heat waves" accompanying headache were reported by 1869 (58.8%) of participants, with 1702 presenting in the previous year and 845 (38.5%) occurring in the majority of attacks. 1245 (38.6%) respondents had visual symptoms that lasted 5–60 min and accompanied headache in the preceding year. This would suggest that criterion B and C for migraine with aura (MwA) was fulfilled, although AMPP questionnaire does not address all MwA ICHD-3 conditions, and consequently a definite diagnosis could not be confirmed. 464 people in this group simultaneously fulfilled diagnostic criteria for MwoA. Sensory symptoms (numbness or tingling) accompanying most severe headache were reported by 2220 (68.8%) of participants.

Warning symptoms before headache onset occurred usually in 1394 (83.0%) of participants with MwoA, while respondents required a median 12 (mean 16.79) hours before feeling normal again after the attack. Sensory stimuli exacerbating headache during migraine attack are shown in Table 2.

**Table 2:** Sensory stimuli usually exacerbating headache or triggering allodynia in MwoA patients

Trigger	*n* (%)
Exposure to heat (examples: cooking; washing face with hot water)	715 (45.6)
Resting face or head on a pillow	635 (39.7)
Combing hair	484 (30.7)
Pulling hair back (for example in a ponytail)	477 (30.9)
Exposure to cold (using an ice pack, washing face with cold water)	369 (23.0)
Wearing eyeglasses	362 (28.5)
Taking a shower (when water jet hits face)	323 (20.8)
Wearing tight clothing	307 (21.5)
Wearing contact lenses	122 (14.1)
Wearing a necklace	109 (8.9)
Wearing earrings	84 (7.05)
Facial shaving (men)	37 (4.64)

### Treatment of migraine

Among MwoA respondents, 1571 (93.6%) had consulted their headache with a medical professional in the past. The first consultation took place when the patients were on average 20.8 years old (median 20). In other words, respondents waited on average 2 years after symptom onset before consulting with a healthcare provider. Neurologists were the most often consulted medical professionals for headache (*n* = 1450 (83.4%). Any primary care physician (general practitioner, internist or paediatrician) had been consulted for headaches by 1393 (82.9%) of respondents with MwoA, while 398 (23.7%) had been seeing one of these professionals on regular basis. The third medical group most often consulted for headache were ophthalmologists (*n* = 987) (58.8). Diagnosis of migraine was confirmed by a medical professional in 1482 (88.3%) MwoA participants. However, this took place on average at the age of 21.4 (median 22), so 4 years after symptom onset, and 2 years after the first headache-related consultation with a medical professional. Other diagnoses received in the past by participants with MwoA have been presented in Table 3.

**Table 3:** Diagnoses received in the past by participants with MwoA

Diagnosis	*n* (%)
Stress-related headache	837 (49.8)
Tension headache	675 (40.2)
Chronic migraine	651 (38.8)
Sinus headache	316 (18.8)
Chronic daily headache	142 (8.5)
Medication-overuse headache	132 (7.9)
Cluster headache	115 (6.8)
Menstrual headache/migraine	48 (28.7)

One thousand five hundred fifty-three (92.5%) of MwoA participants declared their then-current use of some form of treatment. This included abortive physician-prescribed pharmacotherapy (*n* = 1188 (70.8% and over-the-counter medications (*n* = 938 (55.9%). Prophylactic treatment was at some point used by 599 (35.7%) of respondents, while 193 (11.5%) were currently taking preventive medications. Four hundred (23.3%) participants at some point used nutraceuticals for migraine prevention, with magnesium as the most frequently taken supplement.

### Comorbidities

Patients with MwoA have been most often diagnosed with chronic rhinitis (37.1%), allergies (35.9%) and low blood pressure (26.9%). Respondents received on average 8.1 (median 7) points on Patient Health Questionnaire – 9 (PHQ-9). This translated to 65.7% (*n* = 1101) of patients having > 4 points (i.e. depression). Depression was previously diagnosed in 21.3% participants and neurotic/anxiety disorders in 20.4%.

### Burden of migraine

The mean Migraine Disability Assessment (MIDAS) score for participants with MwoA was 42.6 (median 32). 74.9% of respondents stated they required bed rest during attacks, 18.1% had severely impaired ability to work or perform everyday activities, while only 7 (0.4%) declared they could function normally during headache. Migraine-induced disability lasted on average 18.6 h (median 12).

Among MwoA participants, 418 had to resort to sick leave in the two weeks preceding the survey due to health problems (median 2 days per 2 weeks). Within this group, headache was the most prevalent reason for absence (*n* = 329; 78.7%). Moreover, in the two weeks preceding the study, 918 (54.7%) respondents with MwoA went to work despite not feeling well (on median 3 days in this period). The reasons for the above were headache in the case of 793 (86.4%) respondents, followed by back pain (*n* = 141, 15.4%), menstruation (*n* = 118, 12.8%) and depression (*n* = 114, 12.4%).

Respondents attending work with headache required a median of 30 min to start their duties. Even then 78.1% (*n* = 717) did not fulfil their tasks for at least half of the time. Moreover, mistakes made when working with headache were noticed by supervisors, and 24.3% (*n* = 223) of respondents had to spend at least half of their working time on correcting these mistakes. For most of the time the respondents had problems concentrating on their tasks (*n* = 701, 76.4%), performed their duties more slowly (*n* = 730, 79.5%) than usually, or were too exhausted to work (*n* = 618, 67.3%).

On average, participants with migraine spent 292 PLN (median 200) per month on treatment which included mean 187 (median 100) PLN on headache medications and healthcare services.

## Discussion

The Migraine in Poland study is the first large scale survey assessing migraine in this fifth largest European Union member country. This was made possible thanks to the involvement of Polish national mass media, many scientific associations, private and public organizations and social media users. The data collected during the study enables us to describe the sociodemographic characteristics of migraine patients in Poland, the severity of the disease, access to healthcare, treatment patterns, as well as its social, occupational, financial impact and burden of disease.

The vast majority of respondents in this study (87.10%) were women. To some extent this is associated with higher prevalence of migraine among women. Moreover, women are more likely to actively participate in online surveys [14]. A similarly high percentage of women has been reported in other large migraine surveys: 92% among Greek migraine patients [15], or 89.98% in the European Migraine & Headache Alliance (EMHA) survey conducted in various European countries [16]. The distribution of age groups and the average age in our sample, which was 39 years old, were similar to other large survey studies [17–20].

Analyzing the age at which patients first experienced migraine, we found that more than 53% of patients experienced migraine attacks before adulthood. This number was considerably higher than in the Dermitzakis et al. study [15]. However, a population-based study showed similar median age of migraine onset [21]. Early onset of migraine in 31% of patients supports the need to educate medical professionals caring for pediatric population on the proper management of this group of patients [22, 23]. However, our respondents waited on average 2 years before consulting a physician, which indicates that also parents should be the target of education, especially considering that according to our results, it takes on average a further 2 years from the first consultation to the final diagnosis.

MwoA participants had on average 4.69 monthly migraine days and 47.77% of participants had at least 4 migraine days per month. This is the cohort that in most cases should be offered prophylactic treatment. However, only a quarter of this group (*n* = 193) were taking preventive medications. Studies from other countries, including Italy, Japan and the U.S. [24–26], also point out that only small proportion of eligible patients receive prophylactic treatment (9.2–16.8%). Some authors have suggested that it may be due to inexperience of general practitioners in managing patients with frequent migraine attacks [27]. However, in our cohort most patients were consulted by neurologists, hence this problem is not limited to primary care.

Almost 60% of patients reported experiencing visual symptoms in the form of various phenomena in the visual field, and almost 70% of patients reported sensory symptoms. This is a higher percentage than in other studies, in which the observed frequency of visual auras was typically less than 45% and sensory symptoms less than 30% [15, 17, 23]. The explanation of this observation is unclear, and might suggest that prodromal phase symptoms can be in some situations confused with migraine aura; some premonitory phenomena were reported by 83% of respondents. The average postdrome phase lasted 16.8 h, which is in line with results from other studies [15, 18]. The most common sensorimotor stimuli that exacerbate headache include, similar to other observations, exposure to heat and cold, hair brushing, and constant pressure applied to the face [15].

A higher percentage of our participants consulted a medical professional for their headaches (93.57%) than in population-based studies [9, 28]. This seems to be a result of study recruitment method – people with more severe headaches were more likely to take part in the survey than in census studies. Lipton and colleagues analyzed the percentage of specialist consultations of American migraineurs over the years, and demonstrated that it increased steadily –from 16% in 1984 to almost 80% in 2018 [9, 17]. Also of note is that our respondents were consulted mostly by neurologists, unlike in other studies, where general practitioners (GPs) predominated– up to 70% [9, 18, 20, 23]. This difference might be related to the Polish healthcare system organization, which encourages an early consultation with neurologists. It should be noted that over 90% of Poles are entitled to free-of-charge healthcare via national insurance. On the one hand, neurologists in this system can be consulted only after referral by the patient’s GP. On the other hand, there are no mechanisms discouraging GPs from referrals for neurologic consultation. However, waiting time for neurologist consultations in the national healthcare system can be long. This leads to many patients seeking private neurologist consultations.

It is interesting that migraine, despite its high prevalence, is still often misdiagnosed [28–31]. In our group, almost 50% of MwoA respondents received a diagnosis of stress-related headache and more than 40% of tension type headache (TTH) in the past. For comparison, in the MAST study it was 29% and 23%, or in the OVERCOME (Japan) study 15.3% and 18% respectively. Furthermore, in the MAST study, MwoA respondents were less frequently diagnosed with sinus headache (18.8% vs. 36.5%) and cluster headache (6.85 vs. 9.5%) [9, 17] than in our study. Apart from misdiagnosis, this observation might be explained by multiple types of headaches occurring at different times in the same person – in our study, more than 70% of respondents experienced two or more different types of headache. Other studies yielded similar results [30, 32, 33].

The most common MwoA comorbidities included chronic rhinitis, allergies and low blood pressure. These results corroborated with the Japanese study, where allergies accounted for the most common 49.4% of comorbidities [23]. In our cohort, depression was reported by 21.3% participants, which coincides with American observations (23.2%), but is higher than in the Japanese (15.4%) and Israeli (3%) population [23, 34]. A detailed discussion of comorbidities in the Polish Migraine study will be discussed in a separate report.

Moderate or severe migraine-related disability, as assessed by MIDAS scores, was reported by 89.10% of respondents in our study. This is a significantly higher percentage than observed in population-based studies: 20.7% in the Japanese population or 42.5% in the U.S [20, 24]. Moreover, this result is higher than 49.8% observed in the web-based International Burden of Migraine Study (IBMS), which included participants from Australia, Canada, France, Germany, Italy, Spain, Taiwan, the United Kingdom and the United States respectively [35, 36]. This confirms that the vast majority of Poles with migraine experience a significant disability burden. It should also be noted that more than 78% of patients who come to work with a headache use only half of their time at work to perform tasks effectively and nearly 25% make mistakes. This constitutes a significantly higher rate of presenteeism compared to other countries, where it averages 35%-43% [20, 24, 35, 37]. Apart from loss off productivity costs, migraine is also related to a direct economic burden for migraine patients. In our study, the average cost of anti-migraine medications alone was 187 PLN per month, with a minimum national wage of 2200 PLN in 2021 and an average wage of 4249 PLN.

### Limitations

Our study has some limitations. First of these is secondary to the recruitment method – only people with Internet access could take part in our online survey. This would favor respondents from larger municipalities and of higher socioeconomic status – in our survey, more than 48% of respondents were residents of large cities and only 13.3% were countryside residents. Meanwhile, Polish demographic data indicates that about 40% of population lives outside cities. Moreover, only 20.4% of respondents were unemployed or studying: a percentage significantly lower than in the general Polish population (43.8%) [38]. This observation may also indicate higher socio-economic status of our respondents, and it is further supported by the large proportion of our respondents with higher education (55.09% vs 23.1% in general Polish population). Finally, it should be underlined that the invitation to take part in the survey indicated migraine as its main subject. This may have precipitated selection bias, as it can be assumed that people more often suffering from headache would be more willing to participate.

The second limitation results from the retrospective, structured and robust design of the study – patient were asked to recall their complaints. The verification of these findings by prospective headache diaries, headache specialist consultation or diagnostic tests was not possible. Hence the results might be subject to bias related to recollection mistakes, and the misunderstanding of some questions asked in the survey. The omissions were limited by survey construction, which required the respondents to answer all the questions, and which might have precipitated hasty answers in participants weary from having already answered a multitude of questions. However, it should be remembered that these limitations are inevitable in such study design, and have been previously addressed by other authors [9, 10, 20]. It seems that a web-based approach is the appropriate method for collecting cross-sectional or longitudinal data, as it gives access to a larger and more diverse population as compared to a clinical setting [18, 39].

## Conclusions

The Migraine in Poland study fulfills the gaps in knowledge on migraine in Europe. There are many similarities between the results of this study to other large studies from across the world. These similarities include disease symptoms and their significant burden. However, our study points out that migraine management has its individual patterns, secondary to national healthcare system organization. Consequently, solutions targeting migraine management must be tailored at national level. In our opinion, the results of the Migraine in Poland study will help doctors, researchers and decision-makers identify areas of unmet needs, and make informed decisions to improve migraine care in Poland.

## Data Availability

The datasets generated and/or analysed during the current study are available from the corresponding author on reasonable request.

## Abbreviations

AMPP: American migraine prevalence and prevention
BMI: Body mass index
ICHD: International classification of headache disorders
MAST: Migraine in America symptoms and treatment
MHD: Monthly headache day
MIDAS: Migraine disability assessment scale
MwoA: Migraine without aura
OTC: Over-the-counter
PHQ-9: Patient health questionnaire (for Depression and Anxiety)
PLN: Polish zloty
SD: Standard deviation
